# Virulence Characteristics of Carbapenem-Resistant *Klebsiella pneumoniae* Strains from Patients with Necrotizing Skin and Soft Tissue Infections

**DOI:** 10.1038/s41598-017-13524-8

**Published:** 2017-10-19

**Authors:** Fiorella Krapp, Andrew R. Morris, Egon A. Ozer, Alan R. Hauser

**Affiliations:** 1 Department of Medicine, Division of Infectious Diseases, Northwestern University Feinberg School of Medicine, Chicago, Illinois USA; 2 Department of Microbiology-Immunology, Northwestern University Feinberg School of Medicine, Chicago, Illinois USA

## Abstract

Two types of *Klebsiella pneumoniae* (KP) strains are currently emerging: hypervirulent (hvKP) strains and carbapenem-resistant (CR-KP) strains. To date, these two strain types rarely overlap. Recent reports, however, suggest that CR-KP strains are increasing in virulence. hvKP strains frequently present as highly invasive infections, such as necrotizing skin and soft tissue infections (NSSTI). To examine whether CR-KP strains with features of hvKP were present in our U.S. hospital, we retrospectively identified four cases of CR-KP NSSTI diagnosed between January 2012 and January 2016. Whole-genome sequencing was used to perform multilocus sequence typing, capsular typing, and identification of virulence and antimicrobial resistance genes. Additionally, the virulence of each isolate was determined *in vitro* and using murine pneumonia and subcutaneous infection models. We identified one CR-KP isolate that possessed features of hypervirulent KP, including a hypermucoviscous phenotype, K2 capsule, and resistance to phagocytosis. Of the four CR-KP isolates, two had no evidence of enhanced pathogenicity in either mouse model, demonstrating that low-virulence strains can cause NSSTI in immunosuppressed patients. The remaining two isolates exhibited low virulence in the pneumonia model but high virulence in the subcutaneous infection model, suggesting that the virulence attributes of these isolates are adapted to causing NSSTI.

## Introduction

Over the last several decades, *Klebsiella pneumoniae* (KP) has emerged as a cause of community-acquired invasive infections such as necrotizing skin and soft tissue infections (NSSTI), pyogenic liver abscesses, endophthalmitis, and meningitis. The KP strains that cause these infections, referred to as “hypervirulent KP” (hvKP)^1^, were initially noted in Taiwan. Since these early reports, hvKP infections have continued to increase in incidence in Taiwan and have spread across Asia, dramatically changing the epidemiology of many infections in this region of the world. For example, in Taiwan more than 3000 cases of pyogenic liver abscesses now occur each year^2^, of which 80% are caused by KP^3^. In this same country, hvKP causes as many cases of necrotizing fasciitis as *Streptococcus pyogenes* and is associated with a substantially higher mortality (47% vs. 19%)^4^. Importantly, hvKP infections are now being reported around the globe^4–8^, including in North America^7,9^.

hvKP strains appear to be distinct from classical KP (cKP) strains in several microbiological aspects. hvKP isolates frequently have K1 or K2 capsule serotypes^10,11^ and produce large amounts of capsule exopolysaccharide, which results in a hypermucoviscous phenotype when cultured on solid agar. A common feature associated with hvKP is the presence of a large virulence plasmid encoding a number of virulence factors, including a regulator contributing to the hypermucoviscous phenotype (RmpA/A2) and the siderophores aerobactin and salmochelin^12,13^. Additional features associated with hvKP include a ferric uptake operon (*kfuABC)* and an integrative and conjugative element (ICE*Kp1*) encoding the yersiniabactin siderophore system and colibactin^14,15^. Despite the association of particular genetic and phenotypic features with hvKP, studies have noted many exceptions^16,17^. As such, no definitive test to distinguish between cKP and hvKP currently exists.

hvKP isolates have remained generally susceptible to most antibiotics except ampicillin^18,19^. In an early study of community-acquired KP bacteremia (many of which were presumed to be caused by hvKP), only 3.5% of strains produced extended spectrum ß-lactamases (ESBLs)^20^. Coincident with the spread of hvKP has been a rapid and concerning emergence of carbapenem-resistant KP (CR-KP) worldwide. CR-KP strains are now relatively common and pose a frequent therapeutic challenge to physicians. Because patients infected with these strains frequently receive inadequate empiric and definitive antibiotic therapy, they experience mortality rates of 23-75%^21,22^. Fortunately, CR-KP strains have typically been of low virulence and not associated with invasive infections such as NSSTI. In general, these CR-KP isolates have demonstrated low virulence in mouse models of infection and are susceptible to phagocytosis^23^. However, a few cases of NSSTI caused by CR-KP have appeared in the recent literature^24^, raising concerns for the emergence of KP clones that combine multidrug-resistance with a high degree of virulence. This concern is further reinforced by recent case reports of multidrug-resistant hvKP, mostly from Asia^25,26^–including the first cases of carbapenem-resistant hvKP infection^27–29^. To our knowledge, no cases of MDR hvKP have been reported in the U.S. to date. However, highly virulent CR-KP strains may go unrecognized because clinical microbiology laboratories do not routinely screen for the hypermucovisous or hvKP phenotypes. Consequently, virulence characterization of CR-KP strains associated with invasive disease, such as NSSTI, could reveal hypervirulence not detected in CR-KP to date.

We recently identified a case of NSSTI caused by CR-KP at our hospital, a U.S. tertiary care medical center. Since NSSTI is a known manifestation of hvKP, this caused concern that this KP strain might have combined carbapenem-resistance with hypervirulence. To address this concern, we performed a retrospective chart review of all NSSTI cases caused by CR-KP over a four-year period, which identified three additional cases. Here, we describe the clinical, demographic, and antibiotic susceptibility features of these four cases. These four isolates were also tested for a variety of virulence traits *in vitro*, including hypermucoviscosity, capsule production, and phagocytosis resistance. Furthermore, we assessed the *in vivo* virulence potential of each isolate using both pneumonia and subcutaneous murine infection models. From these studies, we identified one CR-KP NSSTI isolate (NU-CRE265) that exhibited several characteristics consistent with hvKP.

## Results

### Clinical characteristics and outcomes of patients diagnosed with CR-KP NSSTI

A retrospective chart review of all cases of KP NSSTI diagnosed at our institution between January 2012 and January 2016 identified four cases of CR-KP NSSTI. The KP isolates from these patients were designated NU-CRE101, NU-CRE176, NU-CRE212, and NU-CRE265. The mean age was 52 years and the mean modified SOFA score was 4 (Table 1). Patients were immunosuppressed (3/4) and/or had diabetes mellitus (2/4). All cases received adequate surgical and antibiotic treatment (Table 1 and Table S1). Although in-hospital mortality was 0%, two patients had relapse of infection requiring readmission, two patients died after discharge, and one of these deaths was a result of the relapse. Thus, the overall-mortality was 50% and the infection-attributable mortality was 25%.

**Table 1:** Demographics, clinical characteristics and outcomes of patients who presented with CR-KP NSSTI.

	NU-CRE101	NU-CRE176	NU-CRE212	NU-CRE265
Clinical Characteristics				
Age, Sex	69, M	50, F	46, M	43, M
Charlson Score	6	4	6	6
Specific Comorbidities	Bladder cancer on chemotherapy	Myasthenia gravis on chronic corticosteroids	DM, CKD on dialysis, failed kidney and pancreas transplant on IS	DM, CKD on dialysis
Immunosuppresed	Yes	Yes	Yes	No
Community-acquired infection	No	Yes	No	No
Post-surgical Infection	No	No	Yes	Yes
Modified SOFA score	2	4	7	4
Fever/Hypotension	Yes/No	No/Yes	Yes/Yes	No/No
Microbiologic characteristics				
Culture source	Tissue	Tissue/Blood	Tissue	Tissue
Polymicrobial culture	VRE	No	MSSA	No
Meropenem Susceptibility	R (>16)	R (>16)	R (>16)	R (>16)
Management				
Source control achieved (surgery)	Yes	Yes	Yes	Yes
Time to effective antibiotic (hs)	39.8	80.7	70.8	131.1
Duration of effective antibiotic (days)	19	unknown^a^	28	unknown^b^
Outcomes				
Length of hospital stay (days)	23	6 (transferred)	13	55
In-hospital death	survived	survived	survived	survived
Relapse	No	after 5 months	after 4 months	No
Overall death (post-discharge)	**Yes**	**Yes**	No	No
Death attributable to infection	No	**Yes**	No	No

DM, diabetes mellitus; CKD, chronic kidney disease; IS, Immunos supressants; VRE, vancomycin-resistant *Enterococcus spp*.; MSSA, methicillin-susceptible *Staphylococcus aureus*.

^a^Total duration of effective antibiotic treatment is unknown, because the patient was transferred to another facility after 6 days of treatment.

^b^Total duration of effective antibiotic treatment is unknown, because the patient was transferred to a rehabilitation facility and had no follow up at our institution.

### Genotypes of CR-KP NSSTI isolates

CR-KP isolates have been associated with globally disseminated clones. To assess the genotypes of the four CR-KP NSSTI isolates, *in silico* multilocus sequence typing (MLST) was performed. Two of the 4 isolates belonged to the globally disseminated MLST group ST258 and one (NU-CRE265) belonged to the ST14 group; ST258 and ST14 have both been previously associated with multidrug-resistant KP outbreaks^30,31^. The fourth isolate belonged to ST1082.

### CR-KP NSSTI isolates carried KPC carbapenemases and a variety of virulence genes

Two main mechanisms of carbapenem resistance have been described among KP strains: production of carbapenemases or production of other ß-lactamases in association with permeability defects of the bacterial cell envelope^32^. In contrast, multiple genes have been associated with increased virulence among KP strains. Whole genome sequencing was used to identify antibiotic resistance determinant genes (including ß-lactamase and carbapenemase genes) carried by the CR-KP strains and to identify virulence genes previously described as associated with hvKP strains. Publicly available databases containing a comprehensive collection of these genes were used for these purposes, as described in the Methods section. As controls, we used the previously characterized low-virulence, non-hypermucoviscous strain MGH78578 and the hypervirulent, hypermucoviscous strain NTUH-K2044. All 4 CR-KP NSSTI isolates carried one carbapenemase gene (*bla*KPC-2 or *bla*KPC-3) in addition to at least one other ß-lactamase gene (Table 2). Virulence genes previously found in hvKP strains were also assessed, including the *rmpA/A2* gene (regulator of mucoid phenotype), fimbrial genes, genes for the biosynthesis or uptake of iron (such as aerobactin, enterobactin, yersiniabactin, and salmochelin)^12–15,26,33^, and for the biosynthesis of the genotoxin colibactin^33^. All CR-KP NSSTI isolates carried type 1 and type 3 fimbrial genes. They also carried genes involved in the synthesis of enterobactin, as well as the aerobactin receptor gene (*iutA*). The presence of other virulence factors associated with iron-acquisition systems (yersiniabactin and *kfuABC*) and colibactin was variable across isolates (Table 2 and Fig. S1).

**Table 2:** Hypermucoviscosity and genomic characterization of CR-KP isolates associated with NSSTI.

	NTUH-2044	MGH78578	NU-CRE101	NU-CRE176	NU-CRE212	NU-CRE265
String test	+	−	−	−	−	+
MLST	ST23	ST38	ST1082	ST258	ST258	ST14
Capsule Serotype	K1	K52	K51	non-typable	non-typable	K2
Virulence factors						
Capsule up-regulation						
rmpA	+	−	−	−	−	−
rmpA2	+	−	−	−	−	−
Siderophore systems						
Enterobactin (*entABCDEF*)	+	+	+	+	+	+
Aerobactin (*iucABCD*)	+	−	−	−	−	−
Aerobactin receptor (*iutA*)	+	+	+	+	+	+
Yersiniabactin (*ybt and irp complex*)	+	−	−	+	+	−
Yersiniabactin receptor (*fyuA*)	+	−	−	+	+	−
Salmochelin (*iroBCD*)	+	−	−	−	−	−
Salmochelin receptor (i*roN*)	+	+	+	+	+	+
Genotoxin						
Colibactin (*clbA* to *clbR*)	−	−	−	+	+	−
Fimbrial genes						
Type 3 fimbrial adhesion genes	+	+	+	+	+	+
Type 1 fimbrial adhesion genes	+	+	+	+	+	+
Other genes						
*kfuABC*	+	−	+	−	−	+
*allABCDRS*	+	−	−	−	−	−
Antibiotic resistance genes						
Beta-lactamases	SHV-11	SHV-11	KPC-2	KPC-3	KPC-3	KPC-3
			SHV-1	SHV-11	SHV-11	SHV-28
				TEM-1	TEM-1A	OXA-9
				OXA-9		
Aminoglycosides resistance genes	−	−	*aadA1*	*aadA1*	*aph(3′ )-Ia*	*aadA1*
			*aac(3)-I*	*aac(6′ )Ib*	*aac(6′)Ib*	*aac(6′ )Ib*
			aacA4	*strA, strB*	*aadA2*	*strA, strB*
Fluoroquinolones resistance genes	*oqxA, oqxB*	*oqxA, oqxB*	*oqxA, oqxB*	*oqxA, oqxB*	*oqxA, oqxB*	*oqxA, oqxB*
			*aac(6′ )Ib-cr*	*aac(6′ )Ib-cr*	*aac(6′ )Ib-cr*	*aac(6′ )Ib-cr*
Other resistance genes	*fosA*	*fosA*	*fosA*, *sul1*,	*fosA*, *sul2*,	*fosA*, *sul1*,	*fosA*, *sul1*,
			*tet(D), dfrA1*	*dfrA14*	*dfrA12, catA1*	*sul2, dfrA14*

MLST, multilocus sequence typing. “+” indicates that gene was present, “−” indicates that gene was absent.

*A complete list of the genes involved in the synthesis of these virulence factors can be found in the Supplementary Figure S1.

### Capsule typing and production

**Figure 1:**
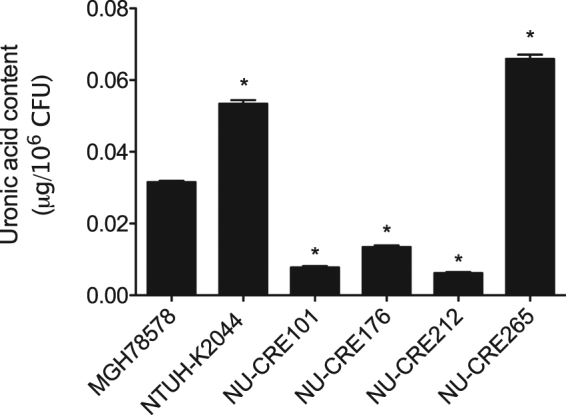
Capsule quantification of CR-KP NSSTI isolates. CPS production was analyzed from equivalent amounts of overnight KP cultures. Total CPS levels were determined by measuring absorbance at 520 nm and normalized to the total viable bacteria (micrograms uronic acid/10^6^ CFU). Data are expressed as means ± SEM. p values were derived from comparisons of each strain to MGH78578 via one-way ANOVA with Bonferroni’s multiple comparison correction (*P ≤ 0.05). Samples were measured in triplicate, and data are representative of three independent experiments.

Capsular serotypes K1 and K2 have been associated with the hypervirulent phenotype^18,34^. To identify the capsule types of the CR-KP isolates in our study, we performed *in silico* capsular polysaccharide (CPS) genotyping using the *wzc* sequence as previously described^35^. A K2 capsular genotype was identified for the CR-KP NSSTI isolate NU-CRE265. The other 3 isolates had a K51 or non-typeable capsular genotype (Table 2). In addition to capsular genotype, hypermucoviscosity and increased capsule production have been associated with highly virulent KP strains^36,37^. We first examined the NSSTI isolates for differences in hypermucoviscosity by the string test method. Only one isolate, NU-CRE265, was found to have a positive string test. We next determined the amount of CPS produced by the NSSTI isolates using an uronic acid quantification assay (Fig. 1). We again used the low-virulence, non-hypermucoviscous strain MGH78578 and the hypervirulent, hypermucoviscous strain NTUH-K2044 as controls. In agreement with previous reports, NTUH-K2044 produced significantly more CPS than MGH78578 (0.053 vs. 0.031 μg/10^6^ CFU, p < 0.05)^38^. Among the NSSTI isolates, only NU-CRE265 produced more capsule than MGH78578 (0.065 μg/10^6^ CFU, p < 0.05 compared to MGH78578). In fact, capsule levels for NU-CRE265 were higher than all the other strains, including NTUH-K2044, consistent with the hypermucoviscous phenotype assigned to this isolate.

### Phagocytic uptake of CR-KP NSSTI isolates

**Figure 2:**
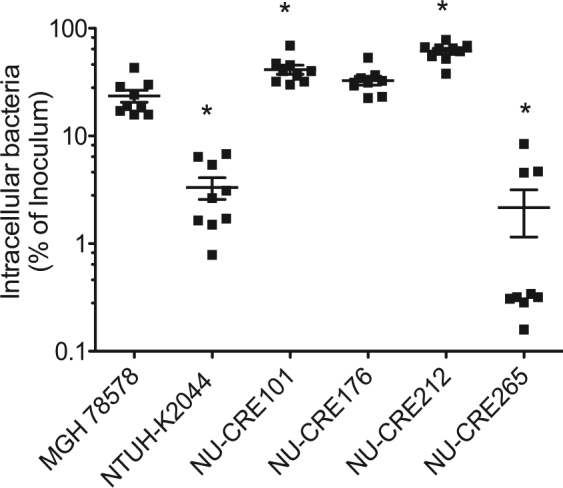
Uptake of CR-KP NSSTI isolates by murine macrophage-like cells. KP strains were incubated with the murine macrophage-like cell line J774.A1 at an MOI of 10. At 1 h post-infection, amikacin was added to the medium, and cells were incubated for 1 h to eradicate extracellular bacteria. The number of intracellular bacteria was then measured by lysing the eukaryotic cells and plating for viable CFUs. The results are expressed as a percentage of the inoculum, and the means and standard deviations are indicated. p values were derived from comparisons of each group to the MGH78578 group via one-way ANOVA with Bonferroni’s multiple comparison correction (*P < 0.05). Each symbol represents the mean of an assay performed in triplicate. Results were combined from three independent experiments.

CPS has been demonstrated to shield KP from phagocytosis and killing by immune cells and in this way contributes to heightened virulence^36^. Indeed, hvKP strains are more resistant to neutrophil-mediated killing than classical KP strains^17^. We evaluated the resistance of the CR-KP NSSTI isolates to phagocytic uptake by the J774 macrophage-like cell line (Fig. 2). Three of the CR-KP NSSTI isolates (NU-CRE101, NU-CRE176, and NU-CRE212) were phagocytosed to a similarly high degree as the low-virulence strain MGH78578. In contrast, both NU-CRE265 and the hypervirulent control strain NTUH-K2044 were highly resistant to phagocytic uptake compared to MGH78578 (p < 0.05). These data are consistent with the high levels of CPS production and the hypermucoviscous phenotype of NU-CRE265 conferring protection from phagocytic uptake.

### Virulence of NSSTI CR-KP isolates in a murine pneumonia model

**Figure 3:**
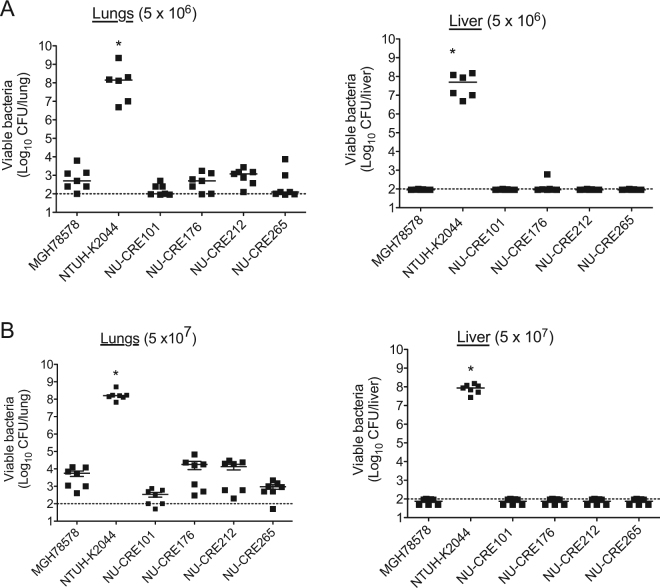
Virulence of CR-KP NSSTI isolates in a mouse model of acute pneumonia. Bacterial burdens within the lungs and livers of C57BL/6 mice intranasally infected with either 5 × 10^6^ (**A**) or 5 × 10^7^ (**B**) CFU of the indicated KP strains were measured. For the highly virulent NTUH-K2044 strain, the inoculum was decreased to 1 × 10^3^ CFU to allow survival out to 96 h post-infection. Organs were harvested and the total viable CFU determined at 96 h post-infection. Each symbol represents the bacterial numbers recovered from a single mouse. Solid bars denote the median CFU, and the dashed line indicates the limit of detection (100 CFU). p values were derived from the comparisons of each group to the MGH78578 infected group (Mann-Whitney U test; *P ≤ 0.05). Data are combined from at least 2 independent experiments (n = 7 for each group).

Although *in vitro* attributes of bacterial isolates are important indicators of their ability to cause severe infections, the true measure of overall virulence is captured by animal models. To investigate the *in vivo* pathogenic potential of the CR-KP NSSTI isolates, we first utilized a murine acute pneumonia model, which has been commonly used to quantify *K. pneumonia* virulence^38^. Mice were infected with either 5 × 10^6^ CFU or 5 × 10^7^ CFU of the CR-KP NSSTI isolates, MGH78578, or NTUH-K2044. Compared to the NTUH-K2044 infected control group, which exhibited 100% mortality by 96 h post-infection, mice infected with the CR-KP NSSTI isolates or MGH78578 exhibited 100% survival out to 14 days post-infection at both tested doses (data not shown). We further examined the bacterial burden in the lungs as well as dissemination to the liver at 96 h post-infection. Consistent with previous reports, the hvKP control strain NTUH-K2044 exhibited proliferation in the lungs and dissemination to the liver^38^ (Fig. 3). In contrast, all the CR-KP NSSTI isolates, as well as the low-virulence strain MGH78578, were cleared from the lungs and failed to disseminate to the liver at both tested doses (Fig. 3). These results indicate that the CR-KP NSSTI isolates have a relatively low level of virulence in a mouse pneumonia model.

### Virulence of NSSTI CR-KP isolates in a mouse subcutaneous infection model

**Figure 4:**
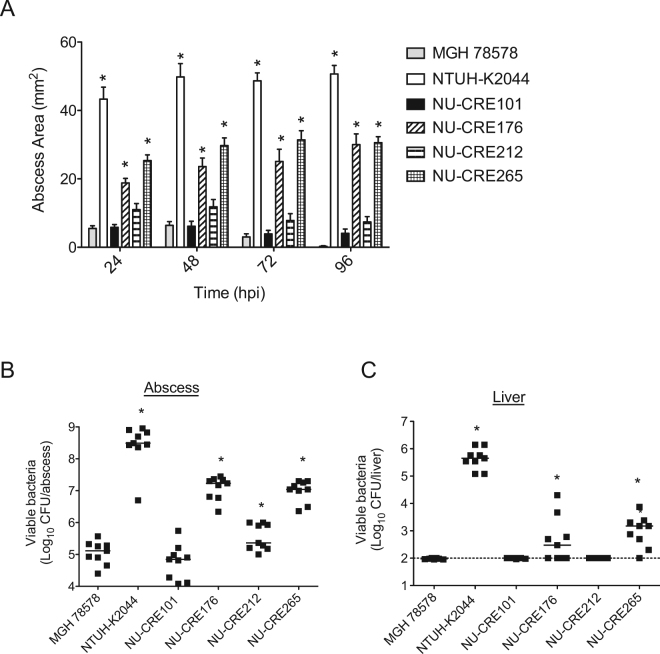
Virulence of CR-KP NSSTI isolates in a subcutaneous infection model. C57BL/6 mice were infected subcutaneously with approximately 5 × 10^6^ CFU of the indicated KP strains. (**A**) Abscess areas were measured up to 96 h post-infection. Data are expressed as means ± SEM. p values were derived from comparisons of each group to the MGH78578 infected group at specified time points (one-way ANOVA with Bonferroni’s multiple comparison correction; *p ≤ 0.05). Bacterial burdens in the abscess (**B**) and the livers (**C**) were determined at 96 h post-infection (hpi). Each symbol represents the bacterial CFU recovered from a single mouse. Solid bars denote the median CFU, and the dashed line indicates the limit of detection (100 CFU). p values were derived from the comparisons of each group to the MGH78578 infected group via the Mann-Whitney U test. *p ≤ 0.05. Data are combined from 3 independent experiments (n = 9 for each group).

Since the CR-KP NSSTI isolates were originally isolated from skin and soft tissue infections, we next investigated the virulence potential of the isolates using a mouse subcutaneous infection model. Mice were infected subcutaneously with 5 × 10^6^ CFU of the CR-KP NSSTI isolates or the control strains MGH78578 and NTUH-K2044, and disease progression was monitored over a 96 h period. In agreement with previous studies, subcutaneous inoculation of KP was found to induce abscess formation within the subcutaneous tissue, which could be quantified by measuring the surface area of the resulting abscess^17,39^. Inoculation with the hypervirulent control strain NTUH-K2044 resulted in the largest abscesses with occasional extension to cause necrosis of the skin, while the low-virulence control MGH78578 induced only small abscess formation and no necrotic lesions (Fig. 4A and Fig. S2). The CR-KP NSSTI isolates varied dramatically in the subcutaneous infection model. NU-CRE265 and NU-CRE176 formed significantly larger abscesses than MGH78578 and the remaining CR-KP NSSTI isolates (Fig. 4A and Fig. S2). In addition to abscess development, we quantified the bacterial burdens within the subcutaneous tissue and livers. Similar to the hypervirulent NTUH-K2044 control, both NU-CRE265 and NU-CRE176 exhibited increased bacterial burdens in the skin and subcutaneous tissue as well as dissemination to the liver at 96 h post-infection (Fig. 4B and C, respectively). These data suggest that both NU-CRE265 and NU-CRE176 exhibit enhanced virulence during skin and soft tissue infection.

Previous reports have demonstrated a critical role for neutrophils in promoting clearance of KP during animal infection^40,41^. Additionally, KP skin infection has been shown to result in the extensive infiltration of neutrophils at the infection site^39^.To further characterize the skin and soft tissue abscesses induced by CR-KP infection, we next examined the host response at 24 h post infection. We performed flow cytometry to quantify the total and specific infiltrating immune cells in the abscess tissue during infection with the virulent NSSTI isolate NU-CRE265. Compared to mock-infected mice, NU-CRE265 infected mice exhibited a robust increase in the total number of immune cells and the number of neutrophils recovered from the abscess tissue (Fig. S3).

**Figure 5:**
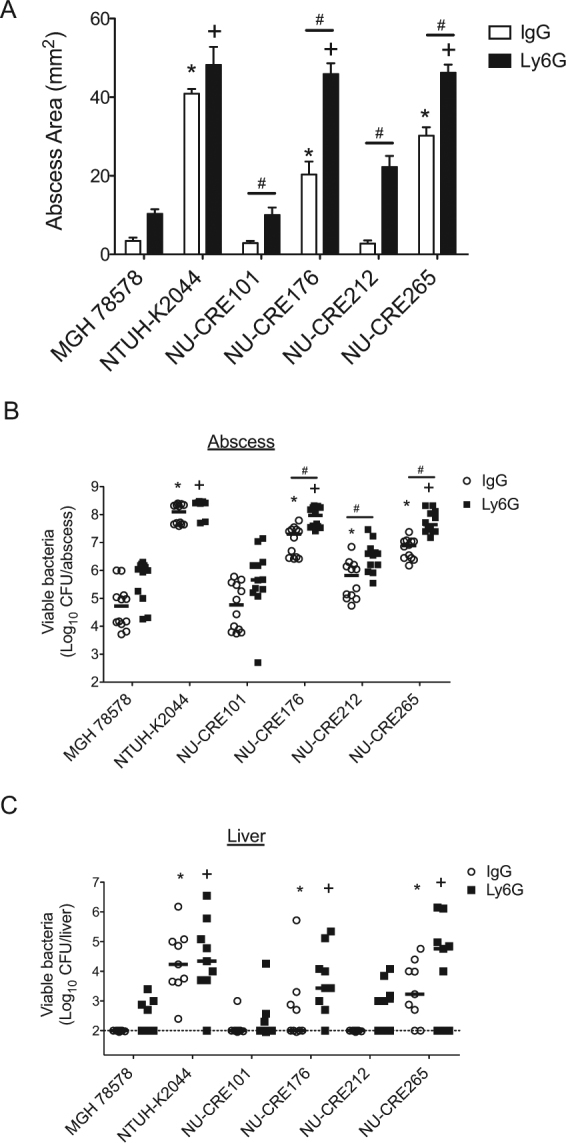
Persistence and dissemination of CR-KP NSSTI isolates following neutrophil depletion in the subcutaneous infection model. C57BL/6 mice treated with either anti-ly6G antibody or IgG isotype control antibody were infected subcutaneously with approximately 5 × 10^6^ CFU of the indicated KP strains. (**A**) Abscess areas were measured at 96 h post-infection. Data are expressed as means ± SEM. Bacterial burdens in the abscesses (**B**) and livers (**C**) were measured at 96 h post-infection. Each symbol represents the bacterial numbers recovered from a single mouse. Solid bars denote the median CFU, and the dashed line indicates the limit of detection (100 CFU). p values were derived from comparisons of each group of mice to the MGH78578 infected groups via one-way ANOVA with Bonferroni’s multiple comparison correction (abscess area) or the Mann-Whitney U test (CFU); *p ≤ 0.05 vs. IgG control group; ^+^p ≤ 0.05 vs. Ly6G treated group. For comparisons within each strain, p values were derived via Student’s *t* test (^#^p ≤ 0.05). Data are combined from 3 independent experiments (n = 9 for each group).

Considering that the CR-KP NSSTI isolates were obtained from immunocompromised patients, we examined whether the potential of these isolates to cause subcutaneous infections was enhanced by neutropenia. Mice were treated with either an isotype control antibody (IgG) or an anti-Ly6G antibody to systemically deplete neutrophils, and then infected subcutaneously with the CR-KP NSSTI isolates or control strains. To confirm the depletion of neutrophils from the Ly6G-treated mice, we performed immune cell staining and flow cytometry on the excised abscess tissue. Ly6G-treated mice exhibited a decrease in total neutrophils and an increase in total macrophages and monocytes in the abscess tissue compared to the IgG-treated control group (Fig. S3). Neutrophil depletion resulted in an overall increase in abscess lesion size (Fig. 5A and Fig. S4), as well as increased bacterial burdens and dissemination to the liver for all CR-KP NSSTI isolates (Fig. 5B and C). However, upon neutrophil depletion, only NU-CRE176 and NU-CRE265 produced abscess lesions comparable to the highly virulent NTUH-K2044 control (48 ± 4 mm^2^ and 46 ± 2 mm^2^ vs. 48 ± 2 mm^2^, respectively; p > 0.05) (Fig. 5A). Additionally, NU-CRE176 and NU-CRE265 exhibited increased bacterial numbers in the abscess (9.3 × 10^7^ and 4.4 × 10^7^ CFU, respectively) and dissemination to the liver (2.7 × 10^3^ and 5.7 × 10^4^ CFU, respectively) at levels similar to the NTUH-K2044 control (abscess = 2.6 × 10^8^ CFU; liver = 2.2 × 10^4^ CFU) (Figs. 5B and C). Interestingly, NTUH-K2044 caused abscesses of similar size and with similar numbers of bacteria regardless of neutropenia (Fig. 5), suggesting that this strain is not affected by the antibacterial functions of neutrophils. Together, these results demonstrate that two of the CR-KP NSSTI isolates, NU-CRE176 and NU-CRE265, exhibit *in vivo* virulence phenotypes similar to highly virulent KP in a subcutaneous infection model. Furthermore, these findings demonstrate the utility of the mouse subcutaneous infection model for identifying and characterizing highly virulent strains of KP and suggest that some KP strains may be adapted to preferentially infect certain tissue types.

### Capsule production is required for KP persistence during skin infection

**Figure 6:**
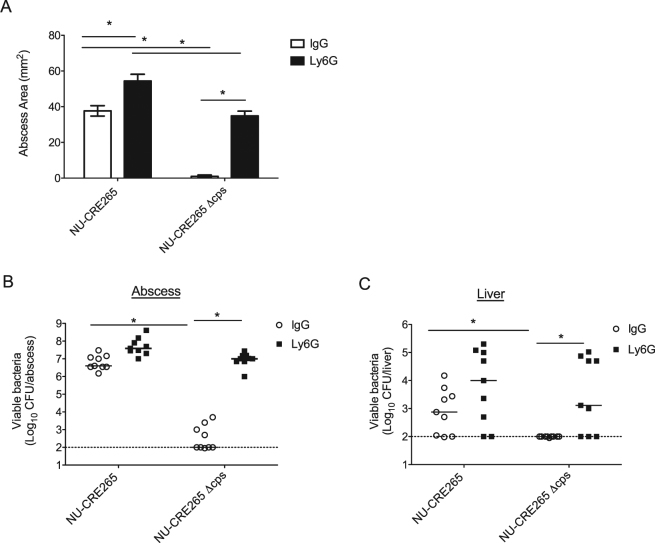
A capsule mutant of NU-CRE265 exhibits impaired virulence in the subcutaneous infection model. C57BL/6 mice treated with either anti-Ly6G antibody or IgG isotype control antibody were infected subcutaneously with approximately 5 × 10^6^ CFU of NU-CRE265 or NU-CRE265*Δcps*. (**A**) Abscess areas were measured at 96 h post-infection. Data are expressed as means ± SEM. Bacterial burdens in the abscesses (**B**) and livers (**C**) were measured at 96 h post-infection. Each symbol represents the number of bacteria recovered from a single mouse. Solid bars denote the median CFU, and the dashed line indicates the limit of detection (100 CFU). *p ≤ 0.05; one-way ANOVA with Bonferroni’s multiple comparison correction (abscess area) or the Mann-Whitney U test (CFU). Data are combined from 2 independent experiments (n = 10 for each group).

Capsule plays an important role in preventing neutrophil-mediated clearance of KP and represents one of the most significant virulence determinants of this bacterium. In a mouse model of KP pulmonary infection, capsule was required for bacterial colonization and persistence^42^. However, the role of capsule in KP pathogenicity during skin and soft tissue infection has yet to be determined. To investigate the impact of capsule production in the subcutaneous infection model, we generated a capsule mutant in the hypermucoviscous and highly virulent NSSTI isolate NU-CRE265. The entire K2 capsule biosynthesis cluster of genes was deleted from NU-CRE265 to generate NU-CRE265*Δcps* (see Supplementary Methods), and the loss of capsule production was confirmed by measurement of the uronic acid content (Fig. S5). We next assessed the sensitivity of the NU-CRE265*Δcps* mutant to phagocytic uptake by J774 macrophage-like cells. As expected, NU-CRE265Δ*cps* exhibited increased phagocytic uptake compared to the parental NU-CRE265 strain (21.1% vs. 1.1% uptake, respectively; p < 0.05) (Fig. S5). To assess the contribution of capsule during skin and soft tissue infection, we subcutaneously infected both neutrophil-replete (IgG isotype control treated) and neutrophil depleted (anti-Ly6G treated) mice with either the parental NU-CRE265 strain or the NU-CRE265*Δcps* mutant. In IgG-treated mice, NU-CRE265*Δcps* infection resulted in dramatically reduced abscess formation at 96 h post-infection (Fig. 6A). NU-CRE265*Δcps* also failed to proliferate within the subcutaneous tissue and to disseminate to the liver in IgG-treated mice (Fig. 6B and C, respectively). In contrast, NU-CRE265Δ*cps* caused large skin abscesses in neutropenic (Ly6G-treated) mice, albeit smaller than the abscesses resulting from infection of neutropenic mice with the parental strain (34.8 ± 7.5 mm^2^ vs. 54.4 ± 9.9 mm^2^, respectively; p > 0.05) (Fig. 6A). Likewise, NU-CRE265*Δcps* bacteria were found in substantial numbers within the abscesses and livers of neutropenic mice at 96 h post infection (Fig. 6B, and C, respectively). In fact, infection of neutrophil-depleted mice with bacteria lacking capsule nicely phenocopied infection of neutrophil-replete mice with wild-type bacteria in abscess size, abscess CFU, and liver CFU. These data indicate that the capsule of KP functions to counteract the sterilizing effects of neutrophils in the mouse subcutaneous infection model.

## Discussion

KP has been recognized as an emerging threat to human health due in part to the rise in multi-drug resistant isolates as well as hypervirulent strains capable of causing invasive infections. To date, these two groups of KP strains have remained largely distinct, but there is concern that strains both highly virulent and highly resistant to antibiotics are emerging. NSSTI are rarely caused by KP but have been identified as one of the clinical manifestations associated with hvKP. Thus, these infections may indicate the presence of hvKP strains. The aim of this study was to identify cases of NSSTI caused by CR-KP among hospitalized patients in a tertiary medical center in the U.S. and to perform a comprehensive evaluation of the virulence of these strains to determine whether they were both CR-KP and hvKP. During the 4-year period, CR-KP caused invasive disease in the form of NSSTI in four patients. A comprehensive virulence assessment demonstrated a range of virulence profiles among the NSSTI CR-KP isolates. While some isolates exhibited low virulence, others demonstrated some features of hvKP such as increased capsule production, resistance to phagocytosis and potential for bacterial dissemination. Moreover, two CR-KP NSSTI isolates were as virulent as a hvKP control strain in the mouse subcutaneous infection model but not in the pneumonia model, suggesting that these strains are adapted to cause more severe skin and soft tissue infections.

We identified one NSSTI CR-KP isolate, NU-CRE265, that exhibited several features previously associated with hvKP. This isolate produced a K2 capsule–a common virulence determinant of hvKP–and was both hypermucoviscous (string test positive) and highly virulent in the mouse subcutaneous infection model. The hypermucoviscosity phenotype was confirmed to be secondary to capsular polysaccharide overproduction. However, the genetic drivers of capsule overproduction in NU-CRE265 appear to differ from those commonly associated other hvKP strains, as this isolate lacked both of the known capsule regulatory genes *rmpA* and *rmpA2*. Hypermucoviscous hvKP strains that lack *rmpA* and *rmpA2* have been previously described, but the mechanisms by which they overexpress their capsules remain unknown^43,44^. NU-CRE265 also carried an intact *kfu* locus (iron uptake system), which has been previously reported in hvKP isolates from pyogenic liver abscesses^15^. However, it lacks other well-characterized virulence factors associated with hvKP, such as allantoin metabolism (the *allS* gene) and the yersiniabactin and aerobactin siderophore systems^45,46^. Of note, 10% and 7–15% of hvKP-like isolates lack yersiniabactin and aerobactin genes, respectively^47–50^, and one study found that the deletion of the genes for yersiniabactin or aerobactin utilization did not impact hvKP virulence in a mouse model^47^. It therefore appears that some hvKP strains have traits that allow them to cause severe invasive infections even in the absence of several of the genes commonly associated with the hypervirulence phenotype. NU-CRE265 may be one of these strains.

In this study, we investigated the virulence potential of the CR-KP NSSTI isolates using mouse pneumonia and subcutaneous infection models. Both these models have been used previously to characterize hvKP strains^38,51^, and pneumonia and NSSTI are both clinical manifestations associated with hvKP^19,20,52^. Intriguingly, while all four CR-KP isolates were relatively avirulent in the mouse pneumonia model, two isolates (NU-CRE176 and NU-CRE265) exhibited virulence phenotypes similar to the hvKP strain NTUH-K2044 in the subcutaneous model. Upon subcutaneous infection, these virulent CR-KP isolates induced the formation of large abscesses and occasional necrotic skin lesions, while also exhibiting increased proliferation within the subcutaneous tissue and enhanced dissemination to the liver. These virulence phenotypes are consistent with those reported in previous studies examining the pathogenesis of KP and other NSSTI-causing pathogens such as *Staphylococcus aureus* in skin and soft tissue infections^39,53,54^. Together, these data suggest that the concept of hvKP may be overly simplified, and that some strains may have the potential to manifest as hvKP in certain tissues but not in others. Indeed, preliminary studies indicate that NU-CRE265 also exhibits low virulence in a mouse intraperitoneal infection model, as indicated by 100% mouse survival upon infection with 5 × 10^6^ CFU of NU-CRE 265 (data not shown), further suggesting that the relative virulence of NU-CRE265 is dependent upon the route or site of infection. The mechanisms underlying the enhanced virulence of the two CR-KP isolates in the subcutaneous infection model compared to the pneumonia model remain unknown. Both NU-CRE176 and NU-CRE265 carried intact loci encoding for type 1 (*fimA* to *fimK*) and type 3 (*mrkABCDF*) fimbrial adhesion genes, and the enterobactin siderophore (*entABCDEF*). While some studies have reported these genes as ubiquitous among clinical KP strains, with prevalence of 95-100%^52,55^, other studies have reported differences in the prevalence based on capsule genotype, hypermucoviscous phenotype and source of infection. For example, *mrkD* has been reported as highly prevalent among K2 strains, hypermucoviscous strains and urine samples, but absent in K1, non-hypermucoviscous strains and respiratory samples^56,57^. In our study, these genes were found in all NSSTI CR-KP isolates. Therefore, it is unlikely that these factors are by themselves responsible for the high virulence in the subcutaneous infection model evidenced only in two of the isolates. While other important virulence factors such as yersiniabactin, colibactin and *kfuABC* were found in half of the NSSTI isolates, they did not explain the difference in virulence seen in the subcutaneous infection model, since they were found in one isolate with high virulence and one with low virulence. Furthermore, other important virulence factors were lacking, such as the capsule regulatory genes *rmpA/A2*, and K1/*magA*, aerobactin, salmochelin, and allantoin metabolism genes. It remains possible that these CR-KP isolates have acquired uncharacterized virulence factors that compensate for the absence of these known virulence factors. Alternatively, several of these hvKP-associated virulence factors may be required for establishing infection in the lungs or dissemination to the liver but may be dispensable during subcutaneous infection^58,59^. Regardless of the explanation, our findings suggest that some KP strains may be better adapted to infect certain tissues than others. Comparative genomics analysis has been performed in the past to detect novel virulence factors^60^. Future studies, with a larger number of strains, could use this approach to compare strains with site-selective virulence and identify site-specific virulence factors.

While the exact set of virulence factors required for hypervirulence in the subcutaneous infection model remains unknown, our studies did identify a critical role for capsule production, as a capsule-defective mutant of NU-CRE265 failed to establish infection and persist within the subcutaneous tissue. The requirement for capsule production during subcutaneous infection was largely dependent on the presence of host neutrophils. In neutropenic mice, the capsule mutant was fully capable of inducing abscess formation and proliferating within the tissue. These findings are consistent with previous studies demonstrating that capsule inhibits KP uptake and clearance by phagocytic cells. Additionally, this work demonstrates the critical role of neutrophils in preventing KP strains from causing NSSTIs. Indeed, even low virulence CR-KP isolates (NU-CRE101 and NU-CRE212) were capable of inducing abscess formation and proliferating in the subcutaneous tissue in neutropenic mice, suggesting that in an immunocompromised host low-virulence strains of KP can cause NSSTIs. These findings suggest that the development of NSSTIs depends upon both the virulence of the KP strain and the immune status of the host. Highly immunocompromised patients may develop NSSTI following infection with even low-virulence KP strains, whereas patients with relatively intact immune systems may develop NSSTI only after exposure to more virulent KP strains. In this regard, it is interesting that NU-CRE265, the strain with features of hvKP, was cultured from the only patient in our series that was not on immune-suppressive therapy (Table 1).

The merging of CR-KP and hvKP is a dreaded development that would result in large numbers of patients with infections that were both severe and difficult to treat. Recent reports indicate that such strains are emerging in Asia^25,26,29^. To date, such strains appear to be absent in the U.S. For this reason, our report of NU-CRE265, a CR-KP strain with features of hvKP, is concerning. Two possible mechanisms for the evolution of these strains are the transfer of plasmids encoding extended-spectrum β-lactamases and carbapenemases to hvKP strains and the horizontal transfer of virulence genes from hvKP to multidrug-resistant KP strains. For example *ybt* genes, which encode for the hvKP siderophore yersiniabactin, can be horizontally acquired^33^; these siderophore genes have recently been noted in a number of carbapenemase-producing ST258 isolates^33^. ST258 strains account for the majority of CR-KP isolates around the globe^61^. One of the strains in our study, NU-CRE212, is an ST258 strain that contained the *ybt* genes (Table 2). Although NU-CR212 had low levels of virulence in both mouse models of infection, the acquisition of *ybt* genes by this strain suggests that CR-KP strains have the potential to acquire virulence genes, a process that could eventually lead to the ability to cause invasive and aggressive infections. These results add to recent reports suggesting that CR-KP strains with increased virulence are emerging and that studies to assess the epidemiology of CR-KP/hvKP are necessary not only in Asia but also in the U.S.

## Methods

### Identification of patients and bacterial isolates

We performed a retrospective study of all cases of CR-KP NSSTI diagnosed among adult patients hospitalized at a tertiary U.S. hospital from January 2012 to January 2016. CR-KP NSSTI cases were defined as skin or soft tissue infection with a necrotizing component confirmed by pathology or by surgical reports *and* tissue cultures positive for KP resistant to at least one carbapenem. For identified cases, the corresponding CR-KP isolates were recovered from a collection of multidrug-resistant isolates archived as part of the routine Institutional Infection Control Policy. NTUH-K2044, a well-characterized hvKP strain, and MGH78578, a well-characterized KP strain with low virulence potential, were used as controls^38^. This study was approved by the Northwestern University Institutional Review Board with a waiver of informed consent due to the retrospective nature of the study. No diagnostic or treatment decisions were affected by this study.

### Clinical and microbiological data

Demographic and clinical data were obtained by chart review. Immunosuppression was defined as receiving chemotherapy or immunosuppressive drugs in the last month. Charlson score and modified sequential organ failure assessment (SOFA) score were used to estimate the level of comorbidities and severity of disease, respectively^62,63^. Adequate surgical treatment was defined as surgical debridement that achieved elimination of necrotic tissue. Antibiotic susceptibility data were determined by Vitek according to CLSI breakpoints^64^. Adequate antibiotic treatment was defined as administration for at least 24 hours of an antibiotic with activity against the cultured KP isolate based on the *in vitro* susceptibility results. Evaluated outcomes included length of hospital stay, relapse, and death. Relapse was defined as the recurrence of a skin or soft tissue infection within 6 months after completion of the antibiotic course at the same site of the initial infection and with a strain of the same species and susceptibility results as the index strain.

### Growth media and culture conditions

KP strains were cultured at 37 °C in Luria-Bertani (LB) broth with shaking or on LB agar. When applicable, LB medium was supplemented with 50 μg/mL apramycin. For Lambda Red mutagenesis, strains were cultured at 30 °C in low salt LB^65^ supplemented with 100 μg/mL of hygromcyin B and 0.1 M L-arabinose.

### Evaluation of hypermucoviscosity

A string test was used to assess for hypermucoviscosity in each CR-KP NSSTI isolate^44^. Briefly, isolates were grown overnight on LB agar. A single colony was lifted with a loop to evaluate for the formation and length of a viscous string between the loop and the colony. A positive string test was defined as a string length ≥ 5 mm. This test was performed twice for each strain for confirmation of the results.

### Measurement of capsule production

Capsule production was measured by quantification of uronic acid extracted from equivalent volumes of overnight cultures, as previously reported^66^. Briefly, extracted samples from 500 μL of overnight cultures were resuspended in water and combined with 1.2 mL sodium tetraborate in concentrated sulfuric acid. Samples were boiled for 5 min, followed by the addition of 20 μL 0.15% 3-hydroxydiphenol in 0.5% NaOH, and the absorbance was measured at 520 nm. CPS levels were determined from a standard curve of D-glucuronic acid (Sigma-Aldrich, St. Louis, MO). Samples were normalized to the total viable bacteria in the culture (micrograms uronic acid/10^6^ CFU) and were measured in triplicate.

### Whole genome sequencing

NSSTI isolates were grown overnight in LB broth with shaking at 37 °C. DNA extraction was performed using Promega Maxwell 16 instrument (Madison, WI). Extracted DNA was processed for DNA library preparation and indexing using the Nextera XT kit (Illumina, San Diego, CA). DNA libraries were then evaluated using the Agilent Bioanalyzer 2100 to determine the DNA fragment size and the Quant-iT dsDNA High-Sensitivity Assay Kit to determine the DNA concentration. Equal amounts of each library were then pooled and run on the Illumina MiSeq system with 300-bp paired-end reads. Raw sequence reads were assembled de novo using SPAdes 3.5.0^67^. Sequencing and assembling were performed blinded to the clinical data and the results of the *in vitro* and *in vivo* assays.

### Molecular typing

Assembled whole genome sequences of the four CR-KP NSSTI isolates were analyzed with the publicly available bioinformatics tool MLST 1.8 (Center for Genomic Epidemiology)^68^ to determine their *in silico* MLST. *In silico* CPS genotyping was performed by aligning the assembled genome sequences of the four NSSTI isolates against a published database of *wzc* sequences linked to capsular serotypes^35^ using BLAST^69^.

### Identification of virulence genes and antibiotic resistance genes

BLAST was used to align de-novo assembled genome sequences against the NCBI Bacterial Antimicrobial Resistance Reference Gene Database (http://www.ncbi.nlm.nih.gov/bioproject/PRJNA313047), ResFinder (http://www.genomicepidemiology.org ^70^), and the Pasteur database of virulence genes (http://bigsdb.pasteur.fr/klebsiella/klebsiella.html). Antibiotic resistance genes investigated included carbapenemases and other ß-lactamase genes. Virulence genes previously associated with hvKP were searched, including hypermucoviscosity-associated genes (*rmpA/A2*); genes associated with iron acquisition systems (aerobactin, yersiniabactin, enterobactin, *salmochelin*, and *kfu*), fimbrial genes (*mrkD*, *fimH*), colibactin genotoxin genes, and allantoin metabolism genes. Thresholds of ≥95% sequence identity and ≥50% gene length were used to detect the target genes. Identified virulence genes were confirmed by aligning each CR-KP NSSTI sequence with NTUH-K2044 and plasmid pK2044 sequences and verifying that the virulence genes detected by our screen had ≥ 95% similarity to the corresponding genes annotated for NTUH-K2044 (Accession number AP006725.1 and AP006726.1).

### Macrophage uptake assay

Resistance to phagocytosis was assessed using a murine macrophage uptake assay^38^. Briefly, J774.A1 macrophage-like cells were cultured in DMEM (Invitrogen, Grand Island, NY) supplemented with heat-inactivated 10% fetal bovine serum and seeded into 24-well microtiter plates at a density of 1 × 10^5^ cells per well. Cells were infected at an MOI of 10 with each KP strain. At 1 h post infection, amikacin (1 mg/mL) was added to the media, and the cells were incubated for 1 h to eradicate extracellular bacteria. Amikacin concentrations were empirically determined to kill extracellular KP in DMEM at >99.99% efficiency within the incubation period. Cell monolayers were washed with PBS, lysed with 0.2% saponin, and the number of intracellular bacteria enumerated by plating for viable CFU. Assays were performed in triplicate.

### Mouse pneumonia model

Respiratory infections were performed as previously described^42^. Mice were anaesthetized via intraperitoneal administration of ketamine (100 mg/mL) and xylazine (20 mg/mL), and then infected with either 5 × 10^6^ or 5 × 10^7^ CFU of each strain diluted in 50 μL of PBS via intranasal administration. Mouse survival was monitored daily up to 14 days. Subgroups of infected mice were euthanized at specific time points (48 and 96 h post-infection) for quantification of bacterial burden in the lungs and the liver. The lungs and livers were excised and homogenized in PBS, and the viable bacteria enumerated by plating serial dilutions. Of note, the inoculum dose for mice infected with the highly virulent NTUH-K2044 strain was decreased to 1 × 10^3^ CFU to allow survival out to 96 h post-infection.

### Mouse subcutaneous infection model

Subcutaneous infections were performed as previously described^39^. Briefly, mice were anesthetized by intraperitoneal injection with a mixture of ketamine and xylazine, shaved in the area of the rear flank, and infected via subcutaneous injection of approximately 5 × 10^6^ CFU of each KP strain diluted in 50 μL PBS. The apparent area of the abscess was quantified daily at the skin surface by multiplying the length of the long and short axes. Subgroups of mice were euthanized at 48 and 96 h post-infection for quantification of bacterial CFU within the abscess and the liver. A standardized surface area of 10 × 10 mm around the initial injection site was excised to a depth of 1 cm. This approach routinely captured the largest abscesses. Livers were aseptically removed from the same mice. Samples were homogenized in PBS, and the viable bacteria quantified by plating serial dilutions.

For neutrophil depletion, mice were injected intraperitoneally with 50 μg of either anti-Ly6G antibody (clone 1A8; BioXCell) or an IgG2A isotype control antibody (2A3; BioXCell) at 1 day prior to infection and again at 1 day post-infection. Mice were infected subcutaneously with approximately 5 × 10^6^ CFU of each KP strain, and the abscess area and bacterial burdens measured.

Animals were purchased from Harlan Laboratories and housed in the containment ward of the Center for Comparative Medicine at Northwestern University. Female C57Bl/6 mice (6- to 10-week-old) were used for all experiments. Experiments were approved by and performed in accordance with the guidelines of the Northwestern University Animal Care and Use Committee.

### Statistical Analysis

Statistical analysis was performed using Student T-test and analysis of variance (ANOVA) followed by the Bonferroni’s correction for multiple comparisons for parametric variables, and the Mann-Whitney U test for non-parametric variables. Statistical significance was defined as p ≤ 0.05.

### Data Availability

The Whole Genome Shotgun projects of the NSSTI CR-KP isolates NU-CRE265, NU-CRE212, NU-CRE176, and NU-CRE101 have been deposited at DDBJ/ENA/GenBank under the accession numbers: NQLL00000000.1, NQLM00000000.1, NQLN00000000.1, NQLO00000000.1, respectively). The datasets and materials generated during the current study are available from the corresponding author on reasonable request.

## Electronic supplementary material

Supplementary information
