# Supplementary material for: Virulence Characteristics of Carbapenem-Resistant Klebsiella pneumoniae Strains from Patients with Necrotizing Skin and Soft Tissue Infections
Source: Sci Rep. 2017 Oct 19;7:13533. doi: 10.1038/s41598-017-13524-8 (PMC5648777; doi:10.1038/s41598-017-13524-8)
Supplement: Supplementary file 1 — Supplementary information [file 41598_2017_13524_MOESM1_ESM.doc]

**Virulence Characteristics of Carbapenem-Resistant *Klebsiella pneumoniae* Strains from Patients with Necrotizing Skin and Soft Tissue Infections**

**Authors**

Fiorella Krapp, MD^1^; Andrew R. Morris, PhD^2^, Egon A. Ozer, MD, PhD^1^, Alan R. Hauser, MD, PhD ^1,2^

^1^ Department of Medicine, Infectious Disease Division, Northwestern University Feinberg School of Medicine, Chicago, Illinois, USA.

^2^Department of Microbiology-Immunology, Northwestern University Feinberg School of Medicine, Chicago, Illinois, USA.

**Corresponding Author**

Fiorella Krapp, MD.

Division of Infectious Diseases, Department of Medicine, Northwestern University

645 N. Michigan Avenue, Suite 900, Chicago, IL  60611, USA

Phone number: 312-695-5090. Fax number: 312-695-5088.

e-mail address: fiorella.krapp@northwestern.edu

Andrew R. Morris, PhD

^2^Department of Microbiology-Immunology, Northwestern University Feinberg School of Medicine, Chicago, Illinois, USA.

Phone number: 312-503-1081 Fax number: 312-503-1339

e-mail address: andrew.morris1@northwestern.edu

**Supplementary Table S1. Antibiotic susceptibility results and antibiotic treatment administered to patients with CR-KP NSSTI**

|  | **NU-CRE101** | | **NU-CRE176** | | **NU-CRE212** | | **NU-CRE265** | |
| --- | --- | --- | --- | --- | --- | --- | --- | --- |
| Antibiotic susceptibility results |  |  |  |  |  |  |  |  |
| Ampicillin/sulbactam | R | >32 | R | >32 | R | >32 | R | >32 |
| Cefazolin | R | >64 | R | >64 | R | >64 | R | >64 |
| Ceftriaxone | R | 8 | R | 32 | R | 8 | R | 16 |
| Cefepime | S | 1 | R | 8 | R | 2 | R | 2 |
| Piperacillin/tazobactam | R | N/P | R | >128 | R | >128 | R | >128 |
| Meropenem | R | >16 | R | >16 | R | >16 | R | >16 |
| Imipenem | R | 4 | N/P |  | N/P |  | N/P |  |
| Aztreonam | R | >64 | R | >64 | R | >64 | R | >64 |
| Amikacin | S | <2 | S | 16 | S | >64 | S | <2 |
| Gentamicin | S | 4 | S | <1 | S | 4 | R | >16 |
| Tobramycin | I | 8 | R | >16 | R | >16 | R | >16 |
| Ciprofloxacin | S | <0.25 | R | >4 | R | >4 | S | 1 |
| TMP/SMX | R | >16/304 | R | >16/304 | R | >16/304 | R | >16/304 |
| Colistin | N/P |  | NI | 0.38 | NI | 0.38 | NI | 1 |
| Tigecycline | N/P |  | NI | 1.5 | NI | 1.5 | NI | 2 |
| Fosfomycin | N/P |  | N/P |  | S | 12 | N/P |  |
| Ceftazidime/avibactam | N/P |  | N/P |  | N/P |  | S | 1 |
|  |  |  |  |  |  |  |  |  |
| Empiric treatment | Pip/tazo | | Pip/tazo | | Pip/tazo | | Pip/tazo |  |
|  | Meropenem | | Cefepime | |  |  | Meropenem | |
|  |  |  | Ciprofloxacin | |  |  |  |  |
| Directed treatment | Ciprofloxacin | | Gentamicin | | Gentamicin | | Tigecycline | |
|  |  |  | Cefepime | | Cefepime | | Ceftaz/avibactam | |
|  |  |  |  |  | Ciprofloxacin | |  |  |
| Time to effective antibiotic (hr) | 39.8 |  | 80.7 |  | 70.8 |  | 131.1 |  |
| Duration of effective antibiotic (days) | 19 |  | unknown | | 28 |  | unknown | |

**R, resistant; I, intermediate; S, susceptible; N/P, not performed; NI, no interpretation available based on CLSI breakpoint guidelines; numbers indicate minimal inhibitory concentrations; Pip/tazo, piperacillin/tazobactam; TMP/SMX, trimethroprim/sulfamethoxazole**

**Supplementary Table S2. Primers used in this study**

| **Primer Name** | **Sequence** |
| --- | --- |
| F-cps-up | 5’ GTGACCGAAATCCCGTAAAC |
| R-cps-up | 5’ GAAGCAGCTCCAGCCTACACATTGTACAAGATCCATTTTCAGC |
| F-cps-down | 5’ GGTCGACGGATCCCCGGAATTAATGCATTACCCATAATGGGAC |
| R-cps-down | 5’ GATCTCTGTGACATCGCGAATAAC |
| ApR-F | 5’ CTTGATATTCCGGGGATCCGTCGACC |
| ApR-R | 5’ TCGATGTGTAGGCTGGAGCTGCTTC |

|  | NTUH-K2044 | MGH78578 | NU-CRE101 | NU-CRE176 | NU-CRE212 | NU-CRE265 | |  |
| --- | --- | --- | --- | --- | --- | --- | --- | --- |
| Enterobactin |  |  |  |  |  |  |  | |
| entA (enterobactin synthase) |  |  |  |  |  |  |  | |
| entB (enterobactin synthase) |  |  |  |  |  |  |  | |
| entC (enterobactin synthase) |  |  |  |  |  |  |  | |
| entD (enterobactin synthase) |  |  |  |  |  |  |  | |
| entE (enterobactin synthase) |  |  |  |  |  |  |  | |
| entF (enterobactin synthase) |  |  |  |  |  |  |  | |
| Aerobactin |  | | | | | |  | |
| iucA (aerobactin synthase) | (p) |  |  |  |  |  |  | |
| iucB (aerobactin synthase) | (p) |  |  |  |  |  |  | |
| iucC (aerobactin synthase) | (p) |  |  |  |  |  |  | |
| iucD (aerobactin synthase) | (p) |  |  |  |  |  |  | |
| iutA (aerobactin receptor) | (p) |  |  |  |  |  |  | |
| Yersiniabactin |  | | | | | |  | |
| ybtA (transcriptional regulator) |  |  |  |  |  |  |  | |
| ybtE (yersiniabactin synthase) |  |  |  |  |  |  |  | |
| ybtP (ABC transporter) |  |  |  |  |  |  |  | |
| ybtQ (ABC transporter) |  |  |  |  |  |  |  | |
| ybtS (salicylate synthase) |  |  |  |  |  |  |  | |
| ybtT (biosynthetic protein) |  |  |  |  |  |  |  | |
| ybtU (biosynthetic protein) |  |  |  |  |  |  |  | |
| ybtX (transporter) |  |  |  |  |  |  |  | |
| irp1 (yersiniabactin synthase) |  |  |  |  |  |  |  | |
| irp2 (yersiniabactin synthase) |  |  |  |  |  |  |  | |
| fyuA (outer membrane receptor) |  |  |  |  |  |  |  | |
| Salmochelin |  | | | | | |  | |
| iroB (glycosyltransferase) | (p) |  |  |  |  |  |  | |
| iroC (ABC transporter) | (p) |  |  |  |  |  |  | |
| iroD (ferric enterochelin esterase) | (p) |  |  |  |  |  |  | |
| iroE (sallmochelin esterase) | (p) |  |  |  |  |  |  | |
| iroN (salmochelin receptor) | (p) |  |  |  |  |  |  | |
| Klebsiella ferric uptake system |  | | | | | |  | |
| kfuA |  |  |  |  |  |  |  | |
| kfuB |  |  |  |  |  |  |  | |
| kfuC |  |  |  |  |  |  |  | |
| Colibactin |  | | | | | |  | |
| clbA (colibactin synthase) |  |  |  |  |  |  |  | |
| clbB (colibactin synthase) |  |  |  |  |  |  |  | |
| clbC (colibactin synthase) |  |  |  |  |  |  |  | |
| clbD (colibactin synthase) |  |  |  |  |  |  |  | |
| clbE (colibactin synthase) |  |  |  |  |  |  |  | |
| clbF (colibactin synthase) |  |  |  |  |  |  |  | |
| clbG (colibactin synthase) |  |  |  |  |  |  |  | |
| clbH (colibactin synthase) |  |  |  |  |  |  |  | |
| clbI (colibactin synthase) |  |  |  |  |  |  |  | |
| clbJ (colibactin synthase) |  |  |  |  |  |  |  | |
| clbK (colibactin synthase) |  |  |  |  |  |  |  | |
| clbL (colibactin synthase) |  |  |  |  |  |  |  | |
| clbM (colibactin synthase) |  |  |  |  |  |  |  | |
| clbN (colibactin synthase) |  |  |  |  |  |  |  | |
| clbO (colibactin synthase) |  |  |  |  |  |  |  | |
| clbP (colibactin synthase) |  |  |  |  |  |  |  | |
| clbQ (colibactin synthase) |  |  |  |  |  |  |  | |
| clbR (colibactin synthase) |  |  |  |  |  |  |  | |
| Type 1 fimbrial genes |  | | | | | |  | |
| fimA |  |  |  |  |  |  |  | |
| fimB |  |  |  |  |  |  |  | |
| fimC |  |  |  |  |  |  |  | |
| fimD |  |  |  |  |  |  |  | |
| fimE |  |  |  |  |  |  |  | |
| fimF |  |  |  |  |  |  |  | |
| fimG |  |  |  |  |  |  |  | |
| fimH |  |  |  |  |  |  |  | |
| fimI |  |  |  |  |  |  |  | |
| Type 3 fimbrial genes |  | | | | | |  | |
| mrkA |  |  |  |  |  |  |  | |
| mrkB |  |  |  |  |  |  |  | |
| mrkC |  |  |  |  |  |  |  | |
| mrkD |  |  |  |  |  |  |  | |
| mrkF |  |  |  |  |  |  |  | |
| Allantoin metabolism |  | | | | | |  | |
| allA |  |  |  |  |  |  |  | |
| allB |  |  |  |  |  |  |  | |
| allC |  |  |  |  |  |  |  | |
| allD |  |  |  |  |  |  |  | |
| allR |  |  |  |  |  |  |  | |
| allS |  |  |  |  |  |  |  | |

**Supplementary Figure S1. The presence in NSSTI CR-KP isolates of genes involved in the synthesis of known virulence factors.** Genes found in each isolate are marked as dark cells. Genes found in the plasmid K2044 are listed as (p). NTUH-K2044, plasmid pK2044 and MGH78578 sequences were downloaded from NCBI (Accession numbers: AP006725.1, AP006726.1 and CP000647.1)

**
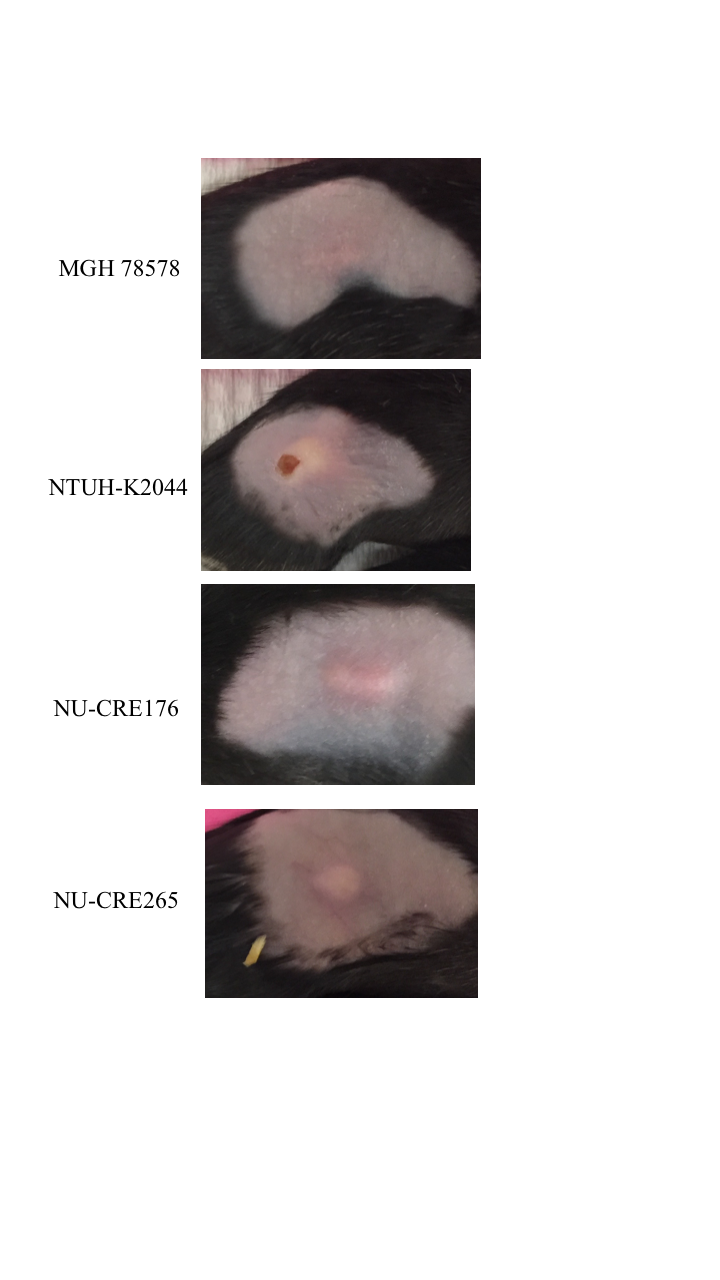
**

**Supplementary Figure S2. Abscess formation during *K. pneumoniae* subcutaneous tissue infection**. C57BL/6 mice were infected subcutaneously with approximately 5 × 10^6^ CFU of the indicated *K. pneumoniae* strains, and abscess formation was monitored. Representative abscess lesion pictures were taken at 96 h post-infection. MGH 78578, low virulence control strain; NTUH-K2044, hypervirulent strain; NU-CRE176 and NU-CRE265, CR-KP NSSTI isolates.

**Supplementary Figure S3. Quantification of immune cell recruitment to abscess tissue following subcutaneous infection with NU-CRE265.** C57BL/6 mice treated with either anti-ly6G antibody (α-Ly6G) or isotype control antibody (α-IgG) were infected subcutaneously with PBS (mock) or approximately 5 × 10^6^ CFU of the virulent NSSTI isolate NU-CRE265. At 24 h post-infection, abscess tissue was excised and collagenase treated, and single-cell suspensions were stained with antibodies for cell surface markers (see Supplementary Methods). (A) Representative flow cytometric plots to confirm depletion of Ly6G-positive neutrophils. The absolute number of CD45+ immune cells (B), neutrophils (C), and macrophages/monocytes (D) were determined within the abscess tissue. Data are combined from 2 independent experiments (n= 6 for each group).

**Supplementary Figure S4. Abscess formation in neutrophil depleted mice.** C57BL/6 mice treated with either anti-ly6G antibody or an IgG isotype control antibody were infected subcutaneously with approximately 5 × 10^6^ CFU of the indicated *K. pneumoniae* strain. The abscess lesion areas were measured over a 96 h time course. Data are expressed as means ± SEM (Student’s *t* test; * p ≤ 0.05). Data are combined from 3 independent experiments (n= 9 for each group). hpi, hours post-infection

**Supplementary Figure S5. Capsule production and phagocytic uptake of the NU-CRE265 capsule mutant**. (A) The levels of CPS production were analyzed from equivalent amounts of overnight cultures of NU-CRE265 and NU-CRE265*Δcps*, the capsule-deficient mutant strain. Total CPS levels were quantified by measuring absorbance at 520 nm, and normalized to total viable bacteria (micrograms uronic acid/10^6^ CFU). Data are expressed as means ± SEM. (B) Phagocytic uptake of NU-CRE265 or NU-CRE265*Δcps* by murine macrophages. *K. pneumoniae* strains were incubated in the presence of the murine macrophage-like cell line J774.A1 at an MOI of 10. At 1 h post-infection, amikacin was added to the media, and cells were incubated for 1 h to eradicate extracellular bacteria. The number of intracellular bacteria was then measured by lysing the J774.A1 cells and plating for viable CFU. The results are expressed as a percentage of the inoculum, and the mean and standard deviation are indicated (Student’s *t* test; * p ≤ 0.05). Samples were measured in triplicate, and data are representative of three independent experiments.

**Supplementary Materials and Methods**

*Construction of NU-CRE265 capsule mutant*

Construction of a capsule-defective mutant (NU-CRE265*Δcps*) was performed using a Lambda Red mutagenesis procedure as previously reported ^1^. Briefly, a deletion construct was generated by PCR amplification of 500 bp sequences upstream of the *wzi* gene and downstream of the *manB* gene within the serotype K2 *cps* region of NU-CRE265 using genomic DNA template and the following primers: F-cps-up and R-cps-up, and F-cps-down and R-cps-down. (All primers are listed in Supplementary Table S1). In a third reaction, the apramycin cassette and flanking FRT sites were PCR amplified using template plasmid pIJ773 and primers ApR-F and ApR-R. The PCR products were purified, pooled, and then joined by overlapping extension PCR using primers F-cps-Up and R-cps-Down. The resulting PCR product was transformed into NU-CRE265 carrying the Lambda Red plasmid pACBSR-Hyg ^1^. The *cps* deletion was confirmed by colony PCR and whole-genome sequencing.

*Skin abscess processing and innate immune cell quantification.*

Mice were infected subcutaneously with NU-CRE265 or mock-infected with PBS, and at 24 h post-infection skin abscesses were harvested. Single-cell suspensions were prepared with collagenase digestion as previously described ^2^. Briefly, a standardized surface area of 10 × 10 mm^2^ around the initial injection site was excised to a depth of 1 cm, the tissue was minced, suspended in RPMI 1640 medium (Invitrogen) containing 0.5% collagenase type I (Sigma-Aldrich, St. Louis, MO), and incubated at 37°C with shaking for 1 h. Single-cell suspensions were obtained by filtering the digested tissue through a 70 μm cell strainer (BD). Cells were resuspended in 2 mL fluorescence-activated cell sorting (FACS) buffer (2% bovine serum albumin in PBS) and total cell numbers determined by Trypan Blue exclusion. For cell surface marker detection, cells were stained with antibodies against CD45 (BD Biosciences; clone 30-F11), GR-1 (eBioscience; clone RB6-8C5), F4/80 (eBioscience; clone BM8), CD11b (BD Biosciences; clone M1/70), and CD11c (BD Biosciences; clone HL3). Antibodies were used at 1:100 dilutions in FACS buffer containing 1:100 dilution of TruStain FcX anti-CD16/32 antibody (BioLegend; clone 93). Samples were analyzed using a BD FACSCanto II flow cytometer and FlowJO software. Identification of cell populations was determined based on the following markers: neutrophils, CD45^+^ F4/80^-^ Ly6G^+^ CD11b^+^, macrophages, CD45^+^ F4/80^+^ Ly6G^-^ CD11b^hi^ CD11c^+^, monocytes, CD45^+^ F4/80^-^ Ly6G^-^ CD11b^+^ CD11c^-^. For confirmation of neutrophil depletion, mice were injected subcutaneously with either anti-Ly6G antibody (1A8) or isotype control antibody 24 h prior to infection with NU-CRE265. At 24 h post-infection, abscess tissue was harvested, digested, and immune cell staining performed as described above.

**References**

1 Huang, T. W. *et al.* Capsule deletion via a lambda-Red knockout system perturbs biofilm formation and fimbriae expression in Klebsiella pneumoniae MGH 78578. *BMC research notes* **7**, 13, doi:10.1186/1756-0500-7-13 (2014).

2 Tkaczyk, C. *et al.* Staphylococcus aureus alpha toxin suppresses effective innate and adaptive immune responses in a murine dermonecrosis model. *PloS one* **8**, e75103, doi:10.1371/journal.pone.0075103 (2013).
